# Supplementary material for: Venomix: a simple bioinformatic pipeline for identifying and characterizing toxin gene candidates from transcriptomic data
Source: PeerJ. 2018 Jul 31;6:e5361. doi: 10.7717/peerj.5361 (PMC6074769; doi:10.7717/peerj.5361)
Supplement: Supplemental Information 3 [file peerj-06-5361-s003.gz › FinalOutput_E-6/Venom_allergen_5/finaltree.pdf]

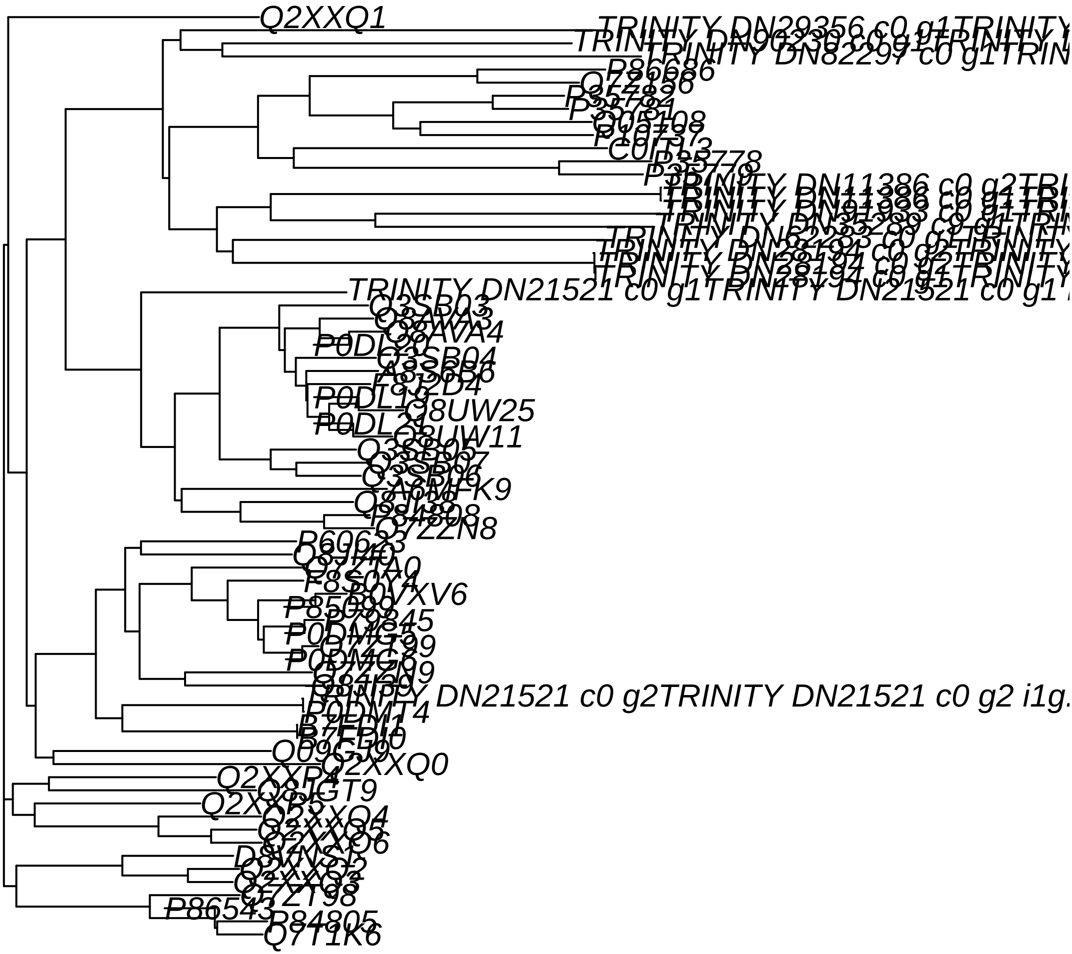
Q2XXQ1
TRINITY DN29356 c0 g1TRINITY
TRINITY DN82297 c0 g1TRIN
P86686
P35784
P35778
TRINITY DN21521 c0 g1TRINITY DN21521 c0 g1
Q3SB03
Q9AVA4
P0DL20
Q3SB04
P0DL19
Q8UW25
P0DL21
Q8UW11
A6MFK9
Q7ZZN8
P60623
Q9YVX6
TRINITY DN21521 c0 g2TRINITY DN21521 c0 g2 i1g.
P0DMT4
Q09GJ9
Q2XXQ0
Q8JGT9
Q2XXQ4
Q2XXQ6
Q7ZT98
P86543
P84805
Q7T1K6
